# Natural Transmission Model for Severe Fever With Thrombocytopenia Syndrome Bunyavirus in Villages of Hubei Province, China

**DOI:** 10.1097/MD.0000000000002533

**Published:** 2016-01-29

**Authors:** Xuesen Xing, Xuhua Guan, Li Liu, Jianbo Zhan, Hongbo Jiang, Li Liu, Guoming Li, Jinfeng Xiong, Liangfei Tan, Junqiang Xu, Yongzhong Jiang, Xuan Yao, Faxian Zhan, Shaofa Nie

**Affiliations:** From Department of Epidemiology and Health Statistics, School of Public Health, Tongji Medical College, Huazhong University of Science and Technology, Wuhan, China (XX, HJ, LL, SN); Hubei Provincial Center for Disease Control and Prevention, Wuhan, China (XX, FZ, XG, LL, JZ, GL, JX, LT, JX, YZ, XY).

## Abstract

Severe fever with thrombocytopenia syndrome (SFTS), an emerging high-fatality infectious disease, is caused by a novel bunyavirus. However, a clear natural transmission model has not yet been established.

We conducted a cross-sectional study with in-depth investigation of villages to systematically understand the transmission and risk factors among humans, host animals, and vectors. Village residents were interviewed using standardized questionnaires, in which there were confirmed cases of new infections, between August 2012 and May 2013. Serum samples from all villagers and animals, as well as tick specimens, were collected for qRT-PCR and antibody testing.

The seropositivity rate among villagers was 8.4% (35/419), which was lower than that among domesticated animals (54.0%, 27/50; *χ*^*2*^ = 81.1, *P <* 0.05). SFTS viral RNA was most commonly detected among domesticated animals (14.0%), followed by ticks (3.1%) and humans (1.7%; *χ*^*2*^ = 23.1, *P <* 0.05). The homology of the S gene fragment was 98%. Tick bites were significantly associated with SFTSV infection (Conditional Logistic Regression odds ratio [OR] = 2.5, 95% confidence interval [CI], 1.0–6.6).

We provided systematic evidence on a natural transmission model for SFTSV from reservoir hosts (domesticated animals) to vectors *(Haemaphysalis longicornis)* to humans, and close contact with SFTS confirmed patients was not found to be a risk factor for natural transmission.

## INTRODUCTION

Severe fever with thrombocytopenia syndrome (SFTS) is a newly identified zoonosis caused by the SFTS bunyavirus (SFTSV), a novel genus of the Bunyaviridae family,^1,2^ with major clinical symptoms of fever, thrombocytopenia, gastrointestinal symptoms, and leukocytopenia. SFTS was first reported among the rural areas of Hubei and Henan provinces in Central China in 2009. To date, SFTS cases have been identified in at least 14 provinces, and they are mainly concentrated in Henan, Shandong, Hubei, Liaoning, Anhui, and Zhejiang. The case-fatality rate of SFTS is up to 30%,^3^ with an average rate of ∼12%.^4^ Cases of SFTS were also reported in Japan and South Korea in 2012, and a disease similar to SFTS has been reported in the United States.^5–7^ SFTSV, and similar viruses, pose an increasingly important threat to global health.

Given the important role of transmission routes in pandemics of infectious diseases, there has been a great deal of research into the transmission model of SFTS. Current evidence concerning the wide detected of corresponding RNA suggests that ticks are the most likely major vectors of the SFTSV. *Haemaphysalis longicornis* from domesticated animals are the dominant species of tick in endemic regions, and SFTSV RNA was isolated from ∼4.9% of the *H. longicornis* specimens collected.^8,9^ The nucleic acid sequences of viruses isolated from ticks have high homology (93%–100%) with SFTSV isolated from patients.^1–3,8–12^ Furthermore, the seasonal distribution of SFTS cases is synchronous with the ecological habits of ticks. Cases start to present around March, peak between May and July, and end around November.^1–3,13–15^ Moreover, a high proportion of patients diagnosed with SFTS report a history of tick bites.^1–3,8,9,16,17^

Domesticated animals may act as amplifying hosts of SFTSV. In the Laizhou and Penglai counties of Shandong Province, 69.5% of sheep, 60.4% of cattle, 37.9% of dogs, and 47.4% of chickens were seropositive for SFTSV.^13^ In Jiangsu Province, 66.8% of goats, 28.2% of cattle, 7.4% of dogs, 4.7% of pigs, 1.2% of chickens, 1.7% of geese, 4.4% of rodents, and 2.7% of hedgehogs were seropositive for SFTSV.^18^ Previous studies have revealed that any potential exposure to ticks, particularly living or working with domesticated animals that present high levels of SFTSV antibodies, including goats, dogs, cattle, pigs, and chickens, increased the incidence rate of SFTS.^13,16–20^ However, only a small proportion of the animals studied (1.7%–5.3%) were found to carry low levels of viral RNA in their sera.^3^

Humans may be a susceptible population. Serosurveillance indicated that 1.0% to 3.8% of the population in hilly areas of China was positive for SFTSV antibodies,^18,21–23^ and SFTSV RNA was not detected in healthy populations. This evidence suggests that people are susceptible to SFTSV. Additionally, the infection may be transmitted from person to person through contact with an infected patient.^3,24,25^

However, these are isolated results, and no systematic field investigations have been performed to illustrate the natural transmission model for SFTS by exploring vectors, hosts, and human populations in a relatively enclosed endemic area; for example, systematic comparisons of SFTSV RNA in ticks, host animals, and human populations, or risk factor analysis based on population exposure. The objective of our study was to systematically illustrate natural infection, a transmission model, and risk factors for SFTSV through in-depth field investigations of villages in a relatively enclosed geographical environment in Hubei Province, China, from August 2012 to May 2013.

## METHODS

### Study Design

**FIGURE 1:**
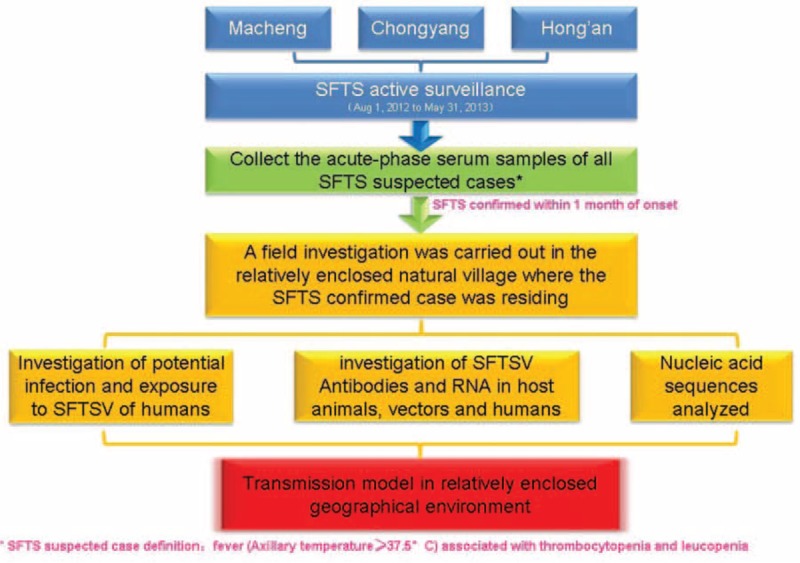
In-depth investigation flowchart of the transmission model in host animals, vectors, and humans.

An active surveillance study was performed in 3 SFTS-endemic counties, including Macheng, Chongyang, and Hong’an, in Hubei Province, China, between August 1, 2012, and May 31, 2013. During this period, the acute-phase serum samples of all outpatients and inpatients who met the diagnostic criteria for SFTS suspected cases were collected and sent to the Hubei Provincial Center for Disease Control and Prevention (HBCDC) for SFTSV quantitative real-time reverse transcription PCR (qRT-PCR) testing. When a case was identified as an SFTS confirmed case within 1 month of onset, an in-depth investigation of potential human infection and exposure to SFTSV and present SFTSV antibodies and RNA in host animals, vectors, and humans was carried out in the relatively enclosed natural villages in which the patients resided (Figure 1).

### Case Definitions of SFTS

As stated in the National Guideline for Prevention and Control of Severe Fever with Thrombocytopenia Syndrome (2010 edition),^26^ an SFTS suspected case is defined as a patient who presents with a fever (auxiliary temperature ≥37.5°C) associated with thrombocytopenia and leucopenia, an SFTS confirmed case is defined as an SFTS suspected case who test positive for virus nucleic acid qRT-PCR or has an antibody titer in the recovery stage that is 4 times higher than in the acute stage or isolated SFTSV. Infected persons are those who are virus nucleic acid qRT-PCR positive or antibody (including IgG and IgM) positive, whereas noninfected persons are those who are virus nucleic acid qRT-PCR and antibody (including IgG and IgM) negative.

### Investigation of Potential Infection and Exposure to Human SFTSV

When a case was confirmed as SFTS within 1 month of onset, all the villagers living in the same relatively enclosed natural village as the patient were interviewed using a standardized questionnaire to collect information on demographic characteristics (eg, age, sex, occupation, residential status, and address); contact with animal (eg, goats, cattle, dogs, pigs, chickens, and rats) blood, secretions, excretions, or saliva within previous month; contact with the saliva, feces, blood, injection site, or skin of patients with confirmed infection within previous month; outdoor activities within previous month (eg, farming, mowing grass, hunting, tea-picking, grazing, and traveling); and history of animal or insect (eg, rats or ticks) bites within the previous month. Blood samples were collected from all the villagers (5 mL per person) on the same day for qRT-PCR and antibody testing (including IgG and IgM) for SFTSV.

### Investigation of Biological Vectors and Host Animals

When a case was confirmed as SFTS within 1 month of onset, the distribution of domesticated (cattle, sheep, etc) and wild (rats, hares, hedgehogs, etc) animals was investigated in the relatively enclosed natural village in which the patient resided. Blood and/or organ samples from all the animals located on the day of the investigation were collected for qRT-PCR and antibody testing (including IgG and IgM) for SFTSV. Meanwhile, the tick distribution in the environment and on the animals was investigated. Tick specimens found on the day of the investigation were gathered to examine qRT-PCR of SFTSV.

### Experimental Detection of SFTSV

TaqMan qRT-PCR was performed on all biological samples using a certified RT-PCR kit for clinical diagnosis (SFDA registration no. 340166, China).^27^ Serological antibodies (including IgG and IgM) against SFTSV were detected using a double-antigen sandwich ELISA.^21^ The DNAStar software SeqMan was used to carry out manual assistance for nucleic acid sequences, and MegAlign software was applied to compare homology. The nucleic acid sequences were compared and analyzed using GenBank, and the nucleic acid sequence alignment analysis was completed using DNAStar 7.

### Statistical Analysis

Pearson chi-square and Fisher's exact tests were applied for proportional comparisons when appropriate. A conditional logistic regression model and the Wald test were used to calculate the maximum likelihood estimates for odds ratios (ORs) and the corresponding 95% confidence intervals (CIs). All tests were 2-tailed, with *P* < 0.05 considered to be statistically significant. SPSS version 12.0 (IBM, Armonk, NY) was used for all statistical analyses.

### Ethical Approval

The collection of data from SFTS patients as part of a continuing public health investigation into an emerging outbreak was authorized by the National Health and Family Planning Commission. The study was approved by the institutional review board of the Hubei Provincial CDC. Written informed consent was obtained from all the participants.

## RESULTS

### Characteristics of Patients With Confirmed Infection

Between August 1, 2012, and May 31, 2013, 7 cases of SFTS were confirmed within a month of onset in 7 relatively enclosed natural villages in Macheng County, Chongyang County, and Hong’an County, Hubei Province. Of the 7 patients, 5 were men and 2 were women, aged 49 to 60 years. All 7 patients were farmers. Two of the patients died. The median interval from onset to clinical diagnosis was 9 days (3–18 days), and the interval from onset to laboratory confirmation was 17 days (8–26 days).

### SFTSV Infection Exposure

Univariate analysis showed that “tick bites,” “contact with animal body fluid,” and “contact with ticks” within the previous month were risk factors for SFTS. “Tick bites” were associated with the highest risk of SFTSV infection (crude OR = 3.0, 95% CI, 1.3–7.2). There was no statistically significant difference between the infected and noninfected persons in the distribution of sex, age, occupation, close contact with patients with confirmed infection, contact with another biological vector, bite from another biological vector, or contact with animal blood or bodily fluids within the previous month (Table 1).

**TABLE 1:**
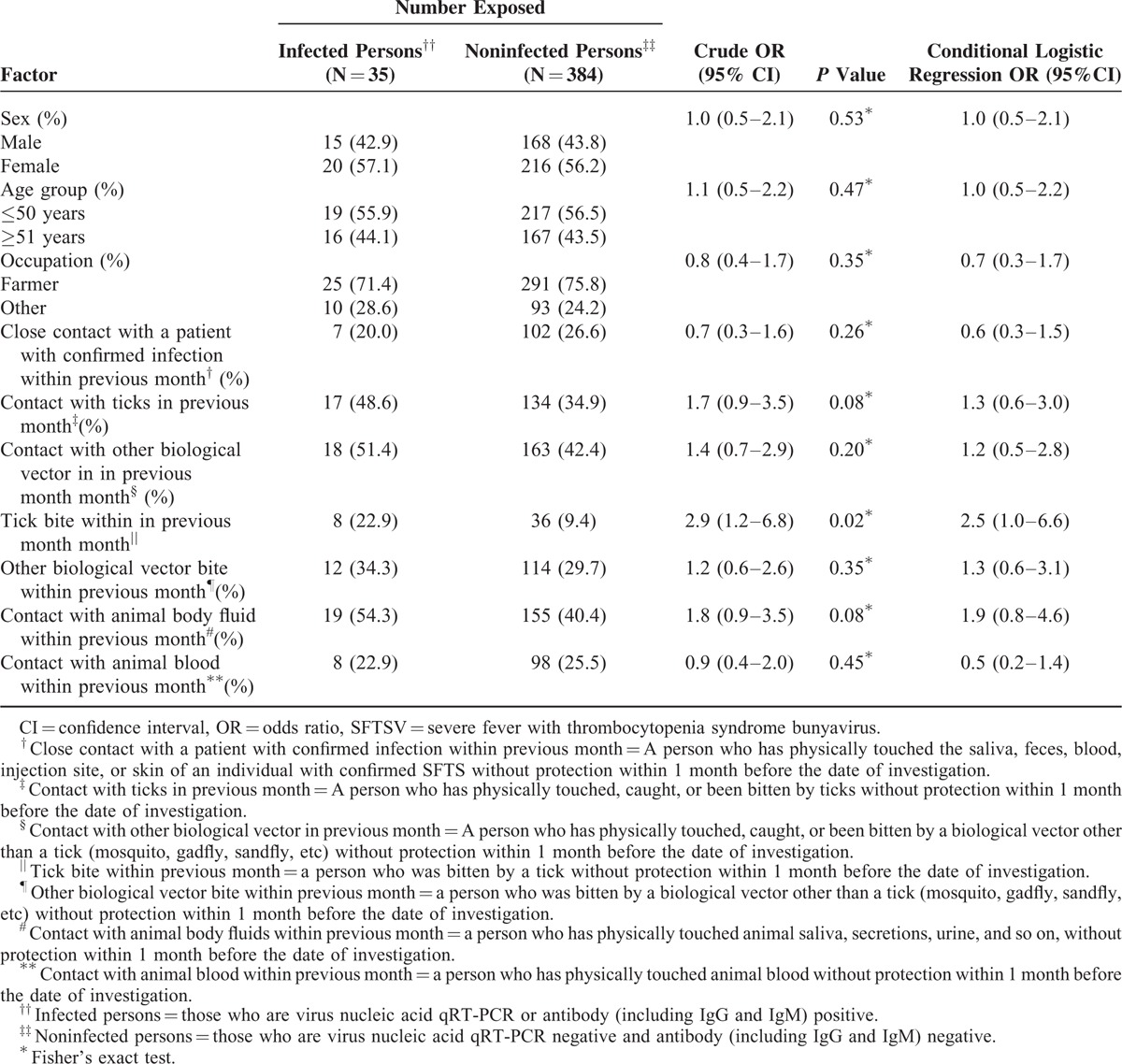
Demographic Characteristics and Exposure Risk Factors Between SFTSV-Infected Persons and Noninfected Persons of 7 Villages in a Relatively Enclosed Geographical Environment in 3 Hubei Province Counties

In multivariable logistic regression analysis of sex, age, occupation, and exposure to patients with confirmed infection, vectors, and animals, only “tick bites within the previous month” were significantly associated with SFTSV infection in humans (conditional logistic regression OR = 2.5, 95% CI, 1.0–6.6) (Table 1).

### SFTSV Antibodies

The rate of seropositivity for SFTSV antibodies in the human population was 8.4% (35/419), which was lower than that observed in domesticated animals (54.0%, 27/50) in the 7 villages (χ^2^ = 81.1, *P* *<* 0.05). Serum antibodies were not detected in any wild animal (0/25).

Among the 50 domesticated animals tested, the seropositivity rate was the highest among cattle (73.7%, 14/19), followed by goats (59.1%, 13/22).

Of the 109 people who reported close contact with the 7 patients with confirmed SFTS, 7 (6.4%) tested seropositive for antibodies. Of the remaining 310 participants, 28 (9.0%) presented positive results for serum antibodies (*P* = 0.4) (Table 2).

**TABLE 2:**
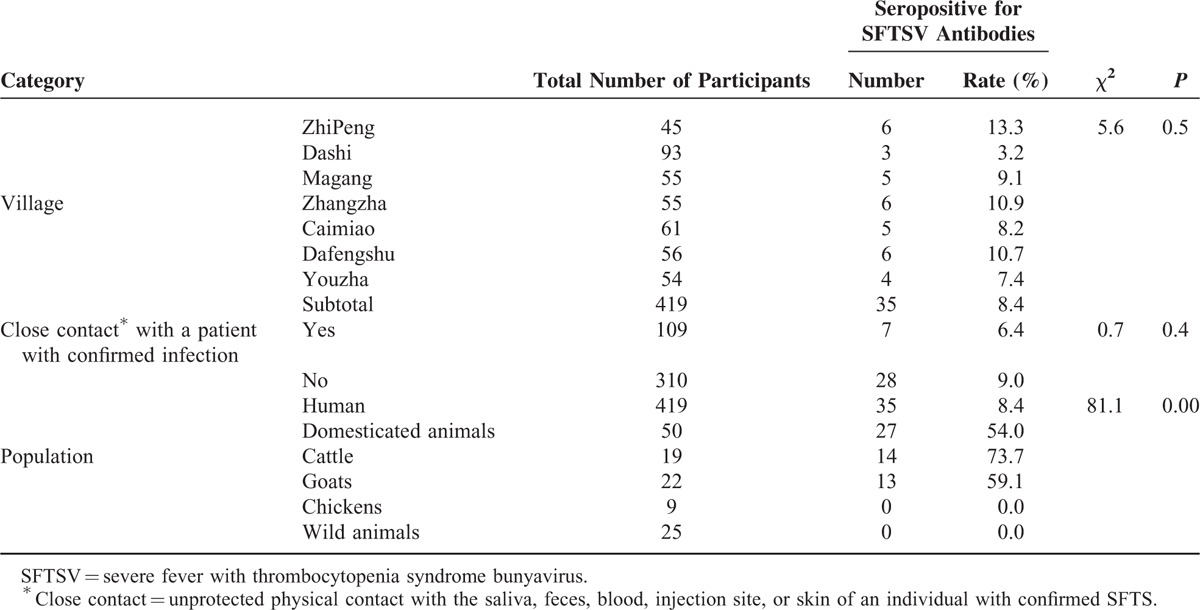
Prevalence of Severe Fever With Thrombocytopenia Syndrome Bunyavirus (SFTSV) Antibodies of 7 Villages in a Relatively Enclosed Geographical Environment in 3 Hubei Province Counties

### SFTSV RNA

In the **7** villages, the positive rate of SFTSV RNA in domesticated animals, ticks, and humans was 14.0% (7/50), 3.1% (8/257), and 1.7% (7/419), respectively (χ^2^ = 23.1, *P* < 0.05) (Table 3). SFTSV RNA was not detected in the wild animals (0/25).

**TABLE 3:**
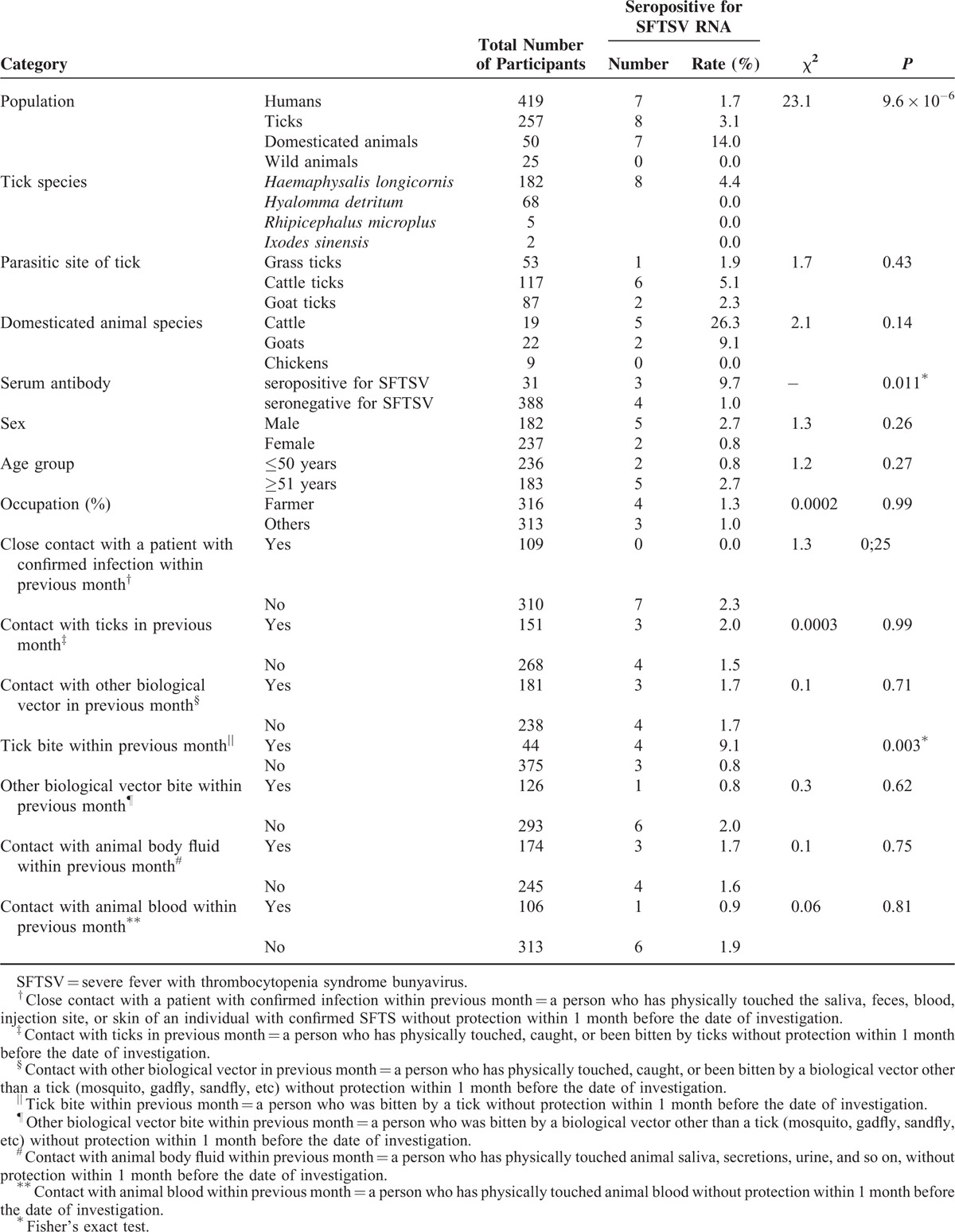
Prevalence of Severe Fever With Thrombocytopenia Syndrome Bunyavirus (SFTSV) RNA of 7 Villages in a Relatively Enclosed Geographical Environment in 3 Hubei Province Counties

Among the 50 domesticated animals tested, the positive rate of SFTSV RNA was highest in cattle (26.3%, 5/19), followed by goats (9.1%, 2/22). Of the 257 tick specimens collected, *H. longicornis* were the dominant species, accounting for 70.8% of all specimens. Furthermore, SFTSV RNA was only detected in *H. longicornis*, with a positive rate of 4.4% (8/182). Analysis of the parasitic animals and their environments indicated that SFTSV RNA positivity was the highest in cattle ticks (5.1%, 6/117), followed by goat ticks (2.3%, 2/87), and then grass ticks (1.9%, 1/53). Of the 419 villagers investigated, only the 7 patients with confirmed infection tested positive for SFTSV RNA (Table 3).

Through comparing RNA positive prevalences among people with different characteristics and epidemiologic histories, we found that the positive rate of SFTSV RNA (9.1%) in persons who were bitten by ticks was significantly higher than the positive rate of SFTSV RNA (0.8%) in persons who were not bitten by ticks (*P* < 0.05) (Table 3).

### SFTSV RNA Sequence Analysis

**FIGURE 2:**
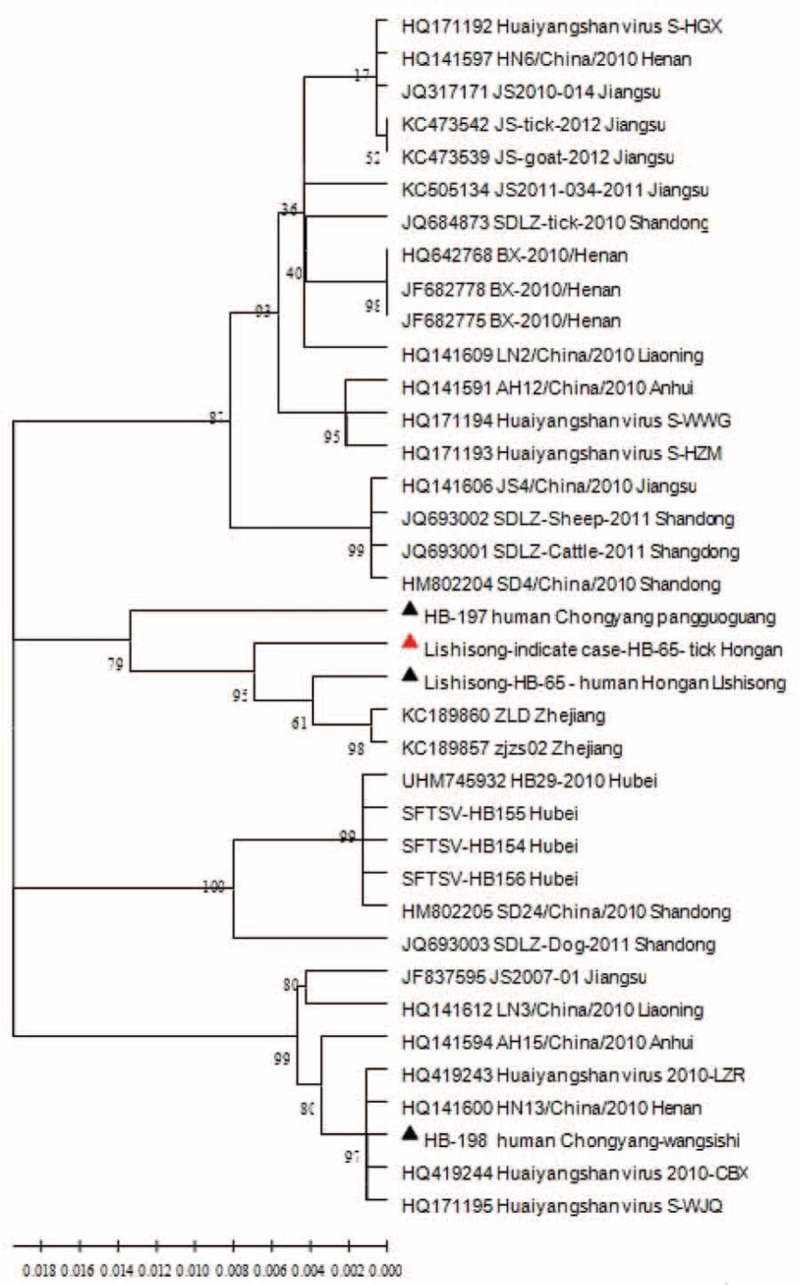
Phylogenetic analysis of severe fever with thrombocytopenia syndrome bunyavirus (SFTSV) isolated from patients with confirmed infection and SFTSV nucleic acid fragments from ticks. SFTSV = severe fever with thrombocytopenia syndrome bunyavirus.

The viral strains were successfully isolated from 3 of the patients with confirmed SFTS, which were named as Lishisong-HB-65-human Hong’an, HB-197 human Chongyang pangguoguang, and HB-198 human Chongyang-wangsishi. An analysis of homology between the 3 SFTSV strains (isolated from the corresponding patients with confirmed infection) and SFTSV nucleic acid fragment from goat ticks from the Lishisong's farm (Lishisong-indicate case-HB-65-tick Hong’an) showed that the homology of the S gene fragment was 98.0% for the viral strains from Lishisong-HB-65-human Hong’an and Lishisong-indicate case-HB-65-tick Hong’an. In addition, the homology of the S gene fragment ranged from 95.8% to 97.9% among Lishisong-HB-65-human Hong’an, HB-197 human Chongyang pangguoguang, HB-198 human Chongyang-wangsishi, and Lishisong-indicate case-HB-65-tick Hong’an (Figure 2).

Furthermore, the homology of the S gene fragment was 98.7% to 98.8% between the Hubei (including Lishisong-HB-65-human Hong’an, HB-197 human Chongyang pangguoguang, HB-198 human Chongyang-wangsishi, and Lishisong-indicate case-HB-65-tick Hong’an) and Zhejiang samples (KC189860 and KC189857). The homology ranged from 95.3% to 96.2% between our collecting samples (Lishisong-HB-65-human Hong’an, HB-197 human Chongyang pangguoguang, and HB-198 human Chongyang-wangsishi) and other strains from Hubei Province including HB29 (JQ416290), HB-155 (JQ733565), and HB-156 (JQ733568). The homology of the S gene fragment was 96.8% for Lishisong-indicate case-HB-65-tick Hong’an and samples of goat ticks from Jiangsu Province, and the homology ranged from 96.3% to 100% for Lishisong-indicate case-HB-65-tick Hong’an and samples of ticks from dogs, sheep, and cattle in Shandong Province (Figure 2).

## DISCUSSION

The results of our in-depth field investigation of 7 villages in a relatively enclosed geographical environment have provided the first systematic evidence for SFTSV natural infection, transmission model, and risk factors from “animals to ticks to humans” in SFTS-endemic counties in Hubei Province, China. We found that the rate of antibody seropositivity in domesticated animals was higher than in humans (χ^2^ = 81.1, *P* *<* 0.05) and was especially high in cattle (73.7%) and goats (59.1%). Moreover, domesticated animals showed the highest positive rate of SFTSV RNA (14.0%), followed by ticks (3.1%), and humans (1.7%) (χ^2^ = 23.1, *P* *<* 0.05). Furthermore, the homology of the S gene fragment was 98% for the SFTSV viral strains isolated from 3 of the patients with confirmed infection and the SFTSV nucleic acid fragment taken from goat ticks. Additionally, we discovered tick bites were the most important exposure risk factor for SFTSV infection in humans (conditional logistic regression OR = 2.5, 95% CI, 1.0–6.6).

The positive rate of SFTSV RNA for ticks collected from cattle and goats ranged from 2.3% to 5.1%, which was higher than that for ticks collected from grass where the cattle and goats grazed (1.9%). SFTSV RNA was not detected in the ticks that were collected from any other animals or environments. These findings indicate that the virus-carrying rate for ticks is related to that in domesticated animals. The positive rate of SFTSV RNA was the lowest in the human population (1.7%). Moreover, SFTSV RNA was not detected in healthy people. The rate of serum antibodies was also the lowest in the human population (8.4%), which indicates that humans are susceptible to SFTS. The positive rate of SFTSV RNA (9.1%) in persons who were bitten by ticks was significantly higher than that in those who were not bitten by ticks (0.8%, *P* < 0.05), thus providing further evidence that tick bites were significantly associated with human SFTSV infection. All 7 of the confirmed patients worked with or had contact with cattle or goats, and the homology of the S gene fragment was 98% for the SFTSV viral strains isolated from 3 of the patients with confirmed infection and the SFTSV nucleic acid taken from goat ticks. In summary, our findings suggest that ticks are the most likely major vectors of SFTSV, and the patients in our study were infected by tick bites. Domesticated animals may act as amplifying and reservoir hosts of SFTSV, and they do not present with symptoms despite high levels of serum antibodies. The animals, particularly cattle and goats, carry SFTSV in their blood and are a major food source for the ticks that spread SFTSV. Previous studies have indicated seropositivity rates for SFTSV RNA in animals of 1.7% to 5.3%, and in humans of 1.0% to 3.8% in Shandong Province and Jiangsu Province.^3,18,21–23^ However, our study showed a higher positive rate of SFTSV RNA (14.0%) and human seroprevalence of SFTSV antibodies (8.4%). These results may be accounted for by our study design, because our field investigations were conducted in relatively enclosed natural villages within 1 month of onset of a confirmed case, compared to the previous studies that did not provide systematic results for animals, vectors, and human populations from the same time and location.

We collected 4 species of tick including *H. longicornis* (70.8%), *Hyalomma detritum* (26.5%), *Rhipicephalus microplus* (1.9%), and *Ixodes sinensis* (0.8%). SFTSV RNA was only detected in *H. longicornis*, with a prevalence of 4.4%. A previous study in Yantai County, Shandong Province, found that from *H. longicornis* (96.9%), *R. microplus* (2.6%), *H. campanulata* (0.3%), and *Dermacentor sinicus* (0.2%), SFTSV RNA was detected in *H. longicornis* and *R. microplus*, with a prevalence of 4.75% for ticks collected from animals and 2.24% for ticks collected from vegetation.^28^ Zhang et al isolated SFTSV RNA from ∼4.9% of the total *H. longicornis* specimens they collected.^8,9^ The homology of the S gene fragment ranged from 96.3% to 100% for tick samples from Hubei, Jiangsu, and Shandong provinces. This suggests that *H. longicornis* is the dominant species in China's endemic regions, and they are the most likely SFTSV vector.

Person-to-person transmission of SFTSV may be possible because the infection has been reported in some family clusters.^3,24,25^ However, we did not find any association between close contact with a patient with confirmed infection. Furthermore, there was no statistically significant difference for seroprevalence of SFTSV antibodies between those who had been in close contact with a patient with confirmed infection and those who had not. These findings suggest that person-to-person transmission is not the main risk factor for infection, and this mode of transmission is probably limited. However, to better understand this mode of transmission, further investigation into the extent of the clinical spectrum of SFTSV-infected individuals, including the extent of subclinical manifestations and the proportion of asymptomatic cases, is required.

The results of our study are subject to several limitations. First, the virus was not isolated in ticks and animals, which limits the ability to generalize the representativeness of SFTS viral diversity in these areas. Second, due to restrictions of the field investigation, the sample sizes for animals and ticks were relatively small. We intend to improve the virus isolation rate and sample representativeness by increasing the sample quantities and improving the collection, preservation, transportation, detection, and culture of the specimens. Third, we did not carry out a cohort study of the viral serum antibody titer and viral load in the host animals and humans, which restricted further study into the mechanisms of SFTSV.

In conclusion, we have provided evidence from a systematic field investigation to show the SFTS transmission model and risk factors among animals, ticks, and humans in 7 villages in a relatively enclosed geographical environment in Hubei Province, China. Domesticated animals, particularly cattle and goats, have high SFTSV infection rates and act as amplifying and reservoir hosts for the virus during the epidemic season in Hubei Province. *H. longicornis* acts as the major vector of SFTSV, and tick bites are the most important risk factor for human infection. Close contact with an individual with confirmed SFTS is not a risk factor for the transmission of infection in humans. Previous studies performed no systematic in-depth field investigations to illustrate natural infection, transmission model, and risk factors for SFTS by exploring vectors, hosts, and human populations, which produced isolated results. Further research is needed to elucidate the natural SFTSV transmission model, including determination of the duration of viremia levels and the possibility of persistent infection in each animal species, potential reservoirs, biological vectors, and the links between SFTSV infection in humans, domesticated animals, and ticks.
